# “No one says ‘No’ to money” – a mixed methods approach for evaluating conditional cash transfer schemes to improve girl children’s status in Haryana, India

**DOI:** 10.1186/1475-9276-13-11

**Published:** 2014-01-31

**Authors:** Anand Krishnan, Ritvik Amarchand, Peter Byass, Chandrakant Pandav, Nawi Ng

**Affiliations:** 1 Centre for Community Medicine, All India Institute of Medical Sciences, New Delhi 110029, India; 2 Umeå Centre for Global Health Research, Department of Public Health and Clinical Medicine, Umeå University, Umeå SE-90187, Sweden

## Abstract

**Introduction:**

Haryana was the first state in India to launch a conditional cash transfer (CCT) scheme in 1994. Initially it targeted all disadvantaged girls but was revised in 2005 to restrict it to second girl children of all groups. The benefit which accrued at girl attaining 18 years and subject to conditionalities of being fully immunized, studying till class 10 and remaining unmarried, was increased from about US$ 500 to US$ 2000. Using a mixed methods approach, we evaluated the implementation and possible impact of these two schemes.

**Methods:**

A survey was conducted among 200 randomly selected respondents of Ballabgarh Block in Haryana to assess their perceptions of girl children and related schemes. A cohort of births during this period was assembled from population database of 28 villages in this block and changes in sex ratio at birth and in immunization coverage at one year of age among boys and girls was measured. Education levels and mean age at marriage of daughters were compared with daughters-in-law from outside Haryana. In-depth interviews were conducted among district level implementers of these schemes to assess their perceptions of programs’ implementation and impact. These were analyzed using a thematic approach.

**Results:**

The perceptions of girls as a liability and poor (9% to 15%) awareness of the schemes was noted. The cohort analysis showed that while there has been an improvement in the indicators studied, these were similar to those seen among the control groups. Qualitative analysis identified a “conspiracy of silence” - an underplaying of the pervasiveness of the problem coupled with a passive implementation of the program and a clash between political culture of giving subsidies and a bureaucratic approach that imposed many conditionalities and documentary needs for availing of benefits.

**Conclusion:**

The apparent lack of impact on the societal mindset calls for a revision in the current approach of addressing a social issue by a purely conditional cash transfer program.

## Introduction

The Millennium Development Goals (MDGs) are ambitious targets aimed at ensuring universal primary education, reducing poverty, combating infectious diseases, and promoting gender equality in the world [1]. Already, the MDGs have catalyzed action at national and international level to unprecedented levels and fostered use of innovative approaches which have helped to lift millions of people out of poverty, save lives and ensure that children attend school, expanded opportunities for women, increased access to clean water and freed many people from diseases [2]. One such new approach is that of use of conditional cash transfer programs (CCTs) [3-5].

CCTs adopt a targeting mechanism; provide cash benefit upon compliance with a set of conditionalities to promote investments in human capital dimensions and, in some cases, also in social capital- such as sending children to school or bringing them to health centers. The success of the first generation of programs in Latin America in increasing enrollment rates, improving preventive health care, and raising household consumption has been documented [6,7]. However, the potential of CCT programs to function well under different conditions and to address a broader range of challenges is not yet clear. For example, such schemes have not been used extensively for addressing the lower status of girls or women in the society. In other words, can CCT programs influence a family to invest more in girls in communities where differences between girls and boys are socially entrenched as in South Asia? The experience in Bangladesh indicates that this works for improving girls’ education but it is not clear whether this resulted in a higher status of girls in the society [8].

Differences in the care of sons and daughters in South Asia stem from parents’ perception of sons as assets and daughters as liabilities [9]. This arises due to perceived differences in the net returns from raising boys and girls. Expected returns of investing in sons are considered higher as males earn higher wages in the labor market and female labor force participation is low. Districts with higher female labor force participation displayed less bias against girls in India [10]. The returns from girls are further compromised because of the practices of dowry and exogamous marriage that effectively reduce girls’ expected contributions to their natal homes while placing sons in the role of providers in old age. Finally, preference for boys is not only for economic reasons but also because of customary and religious practices which put boys on a higher pedestal than girls.

Haryana is one of India’s richest states as measured by per capita GDP, but it ranks among the worst in terms of female disadvantage. In 2011, the lowest sex ratios for those aged 0–6 years were observed in north India, particularly in Haryana (830 girls per 1000 boys) [11]. Studies have also documented a strong pattern of female disadvantage in child survival, health and schooling. We have earlier shown a continuous survival disadvantage for girls from birth to five years of age and shown that this was more common among socio-economically advantaged groups [12,13]. Using a collection of data assembled from the Demographic Health Surveys from a large number of countries including the individual states of India, Filmer et al. report that the *level* of gender disparities in health and education outcomes for girls at the national level in South Asia is the largest in the world, the ratio of female to male child mortality in Haryana is worse than any country in the world, and that there is almost no correlation between per-capita income and the gender disparities in health and education outcomes. So, while the absolute *level* of health and education outcomes for girls are strongly related to economic conditions, the *disparities* between females and males are not [14].

In order to address such big gender gaps, several states in India introduced financial incentive programs in 1990s to discourage son preference among parents and encourage investment in daughters’ education and health. Haryana was the first state in India to launch such a scheme in 1994, which was later replicated in many states. A recent review actually listed fifteen such schemes implemented by the Government of India as well as by state Governments [15]. However, the impact of schemes addressing girl children in India have not been adequately evaluated, primarily owing to their long term nature and short time since their introduction. Most evaluations have focused on process evaluation and included beneficiary and program managers’ feedback [15,16]. The National Advisory Council of Government of India has recommended that such CCT schemes should be fully evaluated [17].

This paper tries to evaluate girl child related schemes being implemented in Haryana State by focusing on one district (Faridabad) using multiple sources of qualitative and quantitative data. The domains of evaluation (data sources) included assessment of community perceptions, program implementers’ perspectives and impact of the program. The Institutional Ethical Committee of the All India Institute of Medical Sciences (AIIMS), New Delhi, cleared the study.

Our quantitative hypotheses were:

1. There is poor awareness and community mobilization on schemes related to girl child in this community.

2. There has been no significant impact of these schemes in terms of a change in sex ratio at birth or higher investment in girl children in the form of higher immunization coverage, education levels and age at marriage as compared to communities where such schemes have not been implemented.

3. To understand the perceptions of the beneficiaries and implementers on the existing girl child schemes and their implementation.

### Description of the schemes

There were two main schemes launched in Haryana as described in Table 1. The first one started in 1994, appropriately labeled as “Apni Beti Apna Dhan” (our daughter, our wealth) to address girls being treated as “paraya dhan” (someone else’s wealth), was aimed at socially disadvantaged populations, with government bonds worth US$ 50 (Indira Vikas Patra) being purchased at the time of birth and the subsequent five years so that beneficiaries would mature with interest to about US$ 500 when the girl became 18 years old. A bonus of US$ 100 is awarded is if the girl has received at least a Standard 5 education, and a further US$ 20 is awarded if she has studied up to Standard 8. In 1995 the Haryana government expanded the scheme by offering a higher maturity amount for girls willing to defer redeeming their securities: US$ 600 for two years, or US$ 700 for 4 years. In addition, they would also receive a credit subsidy for entrepreneurship loans.

**Table 1:** Summary of conditional cash transfer schemes for girl children in Haryana State

**Scheme**	**Apni Beti Apna Dhan (Our daughter, our wealth)**	**LAADLI (Favourite girl)**
Source of funds & year of launch	Government of Haryana	Government of Haryana
	October 1994	August 2005
Beneficiary/conditions Listed	Disadvantaged groups (Scheduled and backward castes and Below Poverty line)	Resident of Haryana on the birth of a second girl child conditional to completion of immunization and schooling.
Benefits/Penalties	US$ 10 to the mother within 15 days of birth.	US$ 100 per family per year upto 5 years invested in Government Bonds. Given at the age of the second girl attaining the age of 18 (matures to around US$ 2000).
	Bonds of US$ 50 in the name of Child within 3 months to mature to US$ 500 by year 18.	
	In 1995, scheme expanded to offer a higher maturity amount for girls willing to defer redeeming their securities: Rs. US$ 600 for 2 yrs, or US$ 700 for 4 yrs.	
Actual beneficiaries/Achievements	2003-04 – 52,501 mothers enrolled in the state	Haryana – 49,558 in 2007–08 with US$ 5 million expenditure and in Faridabad Dist.- 2239
		Cumulatively up to March 2010–1,03,613 families have benefited and US$ 24 million spent
Comments/Remarks	http://faridabad.nic.in/Administration/women&.htm	http://wcdhry.gov.in/new_schemes_F.htm
		Not for the first girl child
	Prevents early marriage as well.	Prevents early marriage as well. Has been extended for next five years.
	http://www.wcdhry.gov.in/admn_2003-2004.pdf	

This scheme was replaced by “Laadli” scheme in 2005 which restricted the benefit to the second girl child, removed restrictions about being disadvantaged and increased the incentive at maturity to about US$ 2,000. Applicants need to submit four documents (domicile certificate, caste certificate (even though it is not a criterion), birth certificate of both girl children and ration card). Also, to be eligible for claim, beneficiaries have to fulfill conditionalities like completing immunization and schooling (with documentation) and remaining unmarried at 18 years. Both the schemes were implemented through Integrated Child Development Services (ICDS) Scheme whose anganwadi workers were entrusted with the responsibility of identifying the beneficiaries and facilitating paper work. As per the data available in their websites, ABAD scheme registered 52,503 beneficiaries in whole of Haryana in one year 2003–04. Laadli scheme also registered about similar numbers in 2007–08 and in 2010 registered 103,613 families.

## Methodology

The study area was Ballabgarh Block of district Faridabad, where AIIMS runs its two primary health centres (PHCs) and has been the subject of our earlier studies on girl child discrimination [12,13,18]. We used a mixed methods approach.

### Quantitative component

This included collection of primary data through interviews and analysis of secondary datasets. Community perspective was assessed through a survey in eight randomly selected villages of Ballabgarh block. Assuming an awareness level of 50% (maximizes sample size) with an error of 10% and a design effect of 2, we arrived at a sample size of 200. One hundred males and 100 females were interviewed from among the houses which had children aged less than or equal to 18 years. In each village, the first respondent was selected randomly from the centre of the village and 25 consecutive houses were selected moving outwards with males and females being studied in alternate houses. The study tool included questions on the background characteristics of the interviewee, community perceptions on girl child discrimination, its reasons, awareness and utilization of government schemes on girl children. It was pilot tested in non-study villages and then modified accordingly. Data collection was done during February-March 2012.

For impact evaluation, a computerized database of 28 villages falling under these two PHCs was used. The electronic database has been described previously and stores population data since 1992 [19]. In brief, demographic data on the whole population is stored and updated annually including births, deaths, marriages and migrations. Relevant variables like immunization and education are also included. The database does not have information on the utilization of the schemes. The dataset from 1992 to 2010 was divided into four time periods dependent upon the introduction of the Schemes. 1992–1994 served as the baseline as no programs had been yet introduced. 1994–2004 was the period when ABAD Scheme was launched (including its modification in 1999). 2005–2010 served as the period when Laadli scheme was launched. For studying the impact of these schemes on sex ratio at birth and immunization coverage, we used birth cohorts of these four time periods. Boys of the same cohort served as the controls for immunization coverage. The hypothesis was that the improvement in immunization coverage of girl children over time would be steeper as compared to boys.

To evaluate improved investment in education of girls and for postponement of marriages, we used cohorts of elder girls who married out at these time periods in these families. As the objective of the scheme is to change the mindset of the society, this should be seen even currently and not wait for actual beneficiaries to mature until the age of marriage. As there are large boy-girl differences especially in the age of marriage, boys would not serve as good controls. Instead, we used daughters-in-law who come from outside Haryana (where there are no such schemes) as controls and compared their age at marriage and education levels to the daughters of the house at the time of marriage.

Data were analyzed using SPSS version 17.0. We used ANOVA to estimate the significance of changes in mean ages observed during these time periods and chi-square for trend for all proportions.

### Qualitative component

In-depth interviews were conducted among two beneficiaries of ABAD and Laadli schemes and all of the two Block (one urban and one rural) and three village level implementers of the government schemes. The selection of village level respondents was convenience based. The interviews were conducted after taking consent from the officials and were audio taped. The interview guide had open-ended questions on presence of girl child discrimination in the community, effectiveness of the schemes, barriers for their implementation and suggestions for improvement. The interviews were conducted in Hindi, transcribed and translated into English by one of the authors (RA).

Data from in-depth interviews were analyzed using thematic analysis approach that allowed researchers to group codes and categories that are similar into themes that reflect specific patterns in the data. All answers were initially read repeatedly to get a sense of the whole and diverseness of the response. Subsequently the free text was coded into meaningful units grounded in the text. These were subsequently categorized manually through an interactive process between the researchers (RA, AK). The categories were then linked into themes and later corroborated by close scrutiny of the analysis [20,21].

## Results

### Quantitative component

The perceptions of the community regarding status of girl child are described in Table 2. It is evident that girl child discrimination and fetal sex determination were the societal norm and this was largely because girl children were considered an economic liability and someone else’s (husbands’ family) asset. This along with the practice of dowry was identified as the main reason why parents were not ready to invest in their girl children. The girl children were less likely to be breast fed, given full diet and be educated. Because the underlying rationale for discrimination was economic, it was also believed by the community members that this problem was more common among poor and uneducated. Even though the participants felt that not enough was being done by the government, they felt that the situation was generally improving for girls. The awareness of ABAD and Laadli among the community members varied between 9% to 15%.

**Table 2:** Community perceptions about girl child and government schemes on it (n = 200)

**Respondents who**	**Proportion (%)**
Believed that society differentiates in bringing up of boys and girls	47
Believed that the girl child is discriminated because she is considered a “paraya dhan” (Others’ asset)	41
Said that pregnant women is pressurized by her spouse/in laws to go for sex determination	77
Estimated that more than 50% of the families in their villages go for sex determination tests	36
Said that government has not done enough to address the problem	68
Suggested following measures that government can take to address this issue	
• Financial help to the families with girl children	79
• Penalties to those going for sex determination	25
Said that there was an improvement in the attitude towards girl child in last few years	72.5
Were aware of	
• Existence of any girl child related schemes	56%
• Apni Beti Apna Dhan (ABAD)	15%
• Laadli	9%

The data on sex ratio at birth (Table 3) shows that the sex ratio at birth worsened consistently (but not significantly) from 866 to 826 girls per 1,000 boys during the study period. However, there was some improvement for the first-born children but the situation in the subsequent born became worse. Immunization coverage at 12 months of age increased significantly for both boys and girls from around 60% in early nineties to above 95% in the latter time period (2005–2010) indicating a general improvement in immunization coverage. Daughters-in law (DILs) from outside Haryana in these villages were more likely to be educated beyond tenth grade and have higher age at marriage as compared to daughters (Table 3). Both educational levels and mean age at marriage showed a significant increase among both daughters and DILs. When DILs from within Haryana were analyzed separately, similar results were found.

**Table 3:** Assessment of impact of conditional cash transfer based interventions on status of girl children (1992–2010)

**Time periods**		**1992-1994**	**1995-1998**	**1999-2004**	**2005-2010**	**P value for trend**
**Indicator**	**Reference group**	**Baseline**	**Apni Beti Apna Dhan**	**Revised ABAD**	**Laadli scheme**	
Sex ratio at Birth (girls per 1000 boys)	All children (n)	866 (6532)	862 (8480)	845 (12488)	826 (12091)	NS
	First Born	827	912	893	904	NS
	Subsequent Born	896	846	825′	780	NS
Proportion completely immunized by 1 year	Girls born in this period	59.8	70.1	88.8	95.8	.00
	Boys born in this period	62.0	71.5	89.9	97.0	.00
Proportion educated ≥ 10^th^ grade among those married in the years	Daughters (n)	2.5 (1370)	4.4 (2298)	8.6 (2475)	27.9 (2578)	.000
	Non-Haryana DILs* (n)	15.8 (476)	18.5 (750)	25.1 (1354)	41.2 (1669)	.000
Mean age ± S.D. at marriage among those married in the years	Daughters (n)	18.3 ± 2.7 (1370)	18.1 ± 2.9 (2298)	18.7 ± 2.9 (2475)	19.7 ± 2.8 (2578)	.0000
	Non-Haryana DILs* (n)	19.1 ± 2.7 (476)	19.1 + 2.9 (750)	19.4 ± 2.8 (1354)	19.7 ± 2.7 (1669)	.0000

*DIL – daughter in-law.

**Table 4:** Themes categories and subcategories of program implementers’ experiences of implementing conditional cash transfer schemes for girl children in Ballabgarh

**Themes identified**	**Categories**	**Subcategories**
“Conspiracy of silence”	Undervalue the seriousness of problem	Restrict the problem to specific groups (Only those with many girls discriminate; This is not seen in urban areas; seen only in slums due to lack of awareness)
		Complete denial of its presence (Have not seen this problem in my area)
	Overrate the effectiveness of the program	The problem is decreasing
		The program has been very effective
	Passive involvement of community in the program	Lack of awareness about the programs
		Anganwadi workers filling the forms house to house
Clash between the political culture of subsidy and bureaucratic approach of accountability	A culture of subsidies	Nobody says no to free money
		Mainly come forward to claim money, no real change
		Why not give it to first child also
“Politician giveth away but Bureaucracy taketh away”		Presence of other schemes like free education, Kanyadaan etc. which also give free subsidies
	Bureaucratic approach of the program	Too many conditionalities
		Need for documentation
		Delay in release of certificates

### Qualitative component

The program implementers had a general assessment that these programs were successful in reducing the girl child discrimination. Increase in registration of birth of girls was reported so that the parents can avail the benefits.

*“Of course, earlier people would not come forward, they would not even tell that a girl child had been born in their house, they would not even celebrate the birth. Because of the scheme they at least come forward and register the birth. Some do this to get benefit of registering for this scheme, some register on their own otherwise too. There are changes, but mostly they come forward (to register) to get these benefits” –* Block level implementer

*“Yes the scheme is good. The main benefit is if there are no girls then how would boys get girls (for marriage)? Earlier people used to practice feticide, but this has now come down – no case in my area till now.”–* Community Level Implementer

A closer scrutiny of the data revealed two basic themes (Table 4). The first one we labelled as a “Conspiracy of Silence”. There was a general tendency to underestimate the pervasiveness of the problem in the community in the indepth interviews, unlike what was seen in the community survey. This occurred either in the form of complete denial or more likely by restricting the problem to a specific sub group – rural, poor, slums, uneducated etc. It was not seen as a problem of the whole community by anybody. This could also take the form of overrating the effectiveness of the program.

*“It is not that discrimination is too much, it is that there are some families which are reluctant to change their practices”*– Block level implementer

A corollary of this was that the program was in fact being implemented in an underhand and passive sort of way. There was no community participation. Local panchayats or health functionaries were not involved in its implementation. Anganwadi workers went house to house and filled the forms of the identified beneficiaries and sent it for registration in the scheme. Even the listed beneficiaries who were interviewed did not know the details of the benefits or conditionalities involved, except that they would come in for some easy money when the girl becomes 18 years old.

*“We don’t know when we get benefits we will see. (After prompts about what they were told when the forms were filled) Yes we will get money after 18 years.” –* Registered beneficiary

The other theme that emerged was a clash between the culture of giving subsidies being promoted by the political class but with the need for accountability by bureaucrats. It appeared that the community’s commitment was to generally get as much subsidy as they could from the government without in any way linking this to any change.

*“No one says ‘no’ to money, all types of people come forward, if they have a daughter they would come for the scheme. When we go to houses……………… we tell them that you will get these benefits then they are more than willing and then we tell them get these papers ready – birth certificates of both girls, domiciles, ration cards and schedule caste certificate”–* Community Level implementer

Too many conditionalities and documentary requirements to access the benefit and delays in getting registered for the schemes also highlighted the problems with the program. The fact that even five to six years after sending the papers, the beneficiaries had not got their saving certificates testifies to a lack of seriousness in the implementation. Also the first year ABAD’s beneficiaries who had their payout scheduled in 2012 (after 18 years); had not received their payments so far.

*In the ratio of 40:60 there would be 60 who do not have the necessary documents, only 40% would be having the relevant documents.–* Community Level Implementer

They also voiced their suggestions that giving the amount at the time of marriage needs to be reviewed. They suggested that the funds can be given earlier so that these are used for girls’ education.

## Discussion

Girl child discrimination as well as use of CCTs as a tool to promote equity by governments have received sufficient attention among academics and policy makers in the recent past. There has been a growing interest in CCTs as a result of their well documented impacts on poverty and inequality as well as on education and health indicators of beneficiaries. Evaluations of CCT in Latin America and South Asia generally show these programs to be effective in raising households’ investment in children [4,6,7]. Most CCT schemes use a well defined and short-term condition (immunization, schooling) for receiving benefits. The schemes evaluated in this paper are different from other CCT programs in both the type of conditionality (daughter’s birth, education and marriage delay) and the long 18-year period over which transfers are made. This also means that a full-impact analysis is not possible currently. Some evaluations have been carried out in the recent past in India of such CCT schemes on girl children [22-27] which have given variable results. Overall our study indicates that while there has been some improvement in the indicators used to measure investment in girl children, these were probably long term trends and were seen among boys as well indicating no specific impact of this scheme in raising the girl children’s status in this community. Our study also raises questions about the program design and implementation.

Haryana pioneered this kind of scheme which has since been replicated in most states of India, and there appears to be a political recognition of the problem. The change from ABAD to Laadli was driven by political reasons as the party in power changed in 2005 and wanted to derive political mileage out of this scheme by launching a new scheme but in reality, tweaking an old one. This means that parties consider such gender related schemes as having a political value, which augurs well for sustainability of such programs in general. These schemes also fit in with the current scenario in India where political parties are vying with each other to dole out subsidies and incentives to win votes. These schemes may not reflect a true commitment of the political class to addressing this issue effectively as this will necessarily pit them against entrenched interests in the community. The subsidy may be seen as an end rather than as a means to an end.

In its first avatar as ABAD, the scheme focused on disadvantaged families and included all girls. This contrasts with the evidence including from Ballabgarh that the practice of sex determination was more among educated and wealthy people [13]. When the scheme was revised, it did away with targeting the disadvantaged but was made applicable only for second girl child. While this change is supported by data, the fact that we focus only on the second child means that the scheme is not looking at changing community’s mindset which needs an universal approach. While many states have kept adoption of a permanent method of contraception as one of the conditionalities, in Haryana subsequent girl children (beyond the second) are not eligible which adds one more dimension to the issue. Conceptually one could also question the need of a program centered around financial incentive on a social problem which is more common among rich and educated and the quantum of benefit being too little for these groups. The maturity amount of US$ 500 would be insufficient for marriage of a girl child even among the poor segments of the population and would be considered too small by the richer segments to effect any behavior change. The cost of marriage in rural Haryana would vary between US$ 10,000 to over US$ 200,000. In addition the cost of raising a child is quite variable and difficult to estimate in rural India, as the investments even for boys are only modest. In the middle income economic group in urban areas, it would be around US$ 100,000 up to 18 years of age. Sekher estimated that the maturity amount was around 3.9% of the investment for child care [15]. People have also questioned the wisdom of bribing parents to keep their daughters thereby reinforcing stereotypes that they are liabilities. Others have also voiced concerns that providing gifts in kind to the girl at the time of her marriage may send out a wrong message to the community as an implicit involvement of the state in covering marriage related financial transactions. Incidentally there is also a government scheme called “Kanyadan” in which disadvantaged people are paid US$ 600 at the time of marriage of their daughters.

Poor involvement of the health department, local governments (*Panchayats*), NGOs, and women’s groups has also been reported by others. Lack of community awareness and participation of panchayats was also reported by Paruthi et al. in a survey among 60 Panchayat members in neighbouring district of Gurgaon of whom only 53% were aware of ABAD [28]. A greater degree of engagement of these stakeholders would result in better monitoring of implementation and also most importantly, place the scheme in a broader context of village or community development.

Our study, as well as previous evaluations of such schemes in other states of India, have highlighted the problems of ambiguous and complicated application processes, and delays in receipt of benefits [16,26]. A common complaint from the beneficiaries across the states has been the difficulty in obtaining various documents required to apply and receive benefits under the schemes, especially the domicile certificates. This calls for simplification of the schemes.

Though year after year substantial financial resources have been directed towards promoting these schemes, there is a lack of field level monitoring. Though random verification is to be carried out by the district level officials to examine the authenticity and eligibility of applicants, lack of field staff means that this was never done. We used our community database to estimate the number of beneficiaries and found out that 13% of births in Ballabgarh villages would qualify as Laadli beneficiaries. As we do not have numbers by rural/urban distribution it is difficult to make an estimate of coverage, but the numbers given indicate moderate coverage.

Both community members and implementers reported that the problem of girl child discrimination was decreasing though this was not supported by data. On the whole, both the government officials and the beneficiaries did recognize some positive aspects of the incentive driven Schemes. A general support for such schemes was noted and has been commented upon by others [15]. The general perceptions of these schemes have been that they are for marriage for children of poor families. As we found, another study reported that only some believed that this was aimed at correcting the sex ratio at birth and very few believed that this was for better education and employability of the girl so as to improve her social status [22].

There are two studies which require closer scrutiny as these have also looked at impact of this scheme in Haryana using secondary data. A World Bank evaluation of ABAD scheme using National Family Health Survey (NFHS) data showed that it had a positive effect on the sex ratio of living children, but inconclusive effects on mothers’ preferences for having female children as well as total desired fertility. They also reported that parents made greater post-natal health investments in eligible girls, though the early cohort of eligible school-age girls were not significantly more likely to attend school. However, conditional on attending any school, they were more likely to continue their education. The survival of girl children improved during the period 1993–2006 at a much higher rate as compared to boys and among girls, the improvement in ABAD eligibles (disadvantaged) was more than that of non-eligibles indicating a positive program impact on survival [29].

Mazumdar evaluated Laadli Scheme using two rounds of District Level Household Survey- 2002–04 (baseline) and 2007–08 (post-intervention) [30]. They used data from Punjab (a neighbouring state which also has gender based discrimination) as a control. A difference-in-difference analysis showed that the increase in percentage of women who had at least one daughter between 2004 to 2008 was not significantly more in Haryana (0.42%). When this analysis was restricted to neighboring districts as being socio-culturally similar (Faridabad got excluded), then the likelihood of having at least one daughter increased significantly by 2.3% between 2004 and 2008 in Haryana as compared to Punjab. The difference was much lower if the sample was restricted to women who already had a girl child (intended beneficiary of Laadli), even in the restricted analysis of neighboring districts. The authors concluded that the improved sex ratio may not be attributable to Laadli Scheme and also raise the possibility that the scheme may not have been uniformly effective in all parts of the state.

The differences in the results of these two studies and our study need careful scrutiny. While both the above mentioned studies included the whole of Haryana and used secondary data that was periodically collected, we restricted our analysis to one block in a district of Haryana but used a better quality longitudinal dataset where all births and deaths have been counted for many decades. The possibility of differential implementation and effectiveness of schemes has already been commented upon. There was only pre-and-post control in the study by the World Bank whereas the other two studies had concurrent controls. Our study used daughters-in-law from outside Haryana as controls while Mazumdar et al. used girls from a neighboring state. We used data up to 2010 which meant a longer time for programs to show impact as compared to others. Increase in girl child registration is a positive fallout of these interventions, and this could result in a spurious increase in reported sex ratio at birth and therefore, using this indicator to measure success of the program needs caution. Earlier rounds of NFHS have been criticized for underreporting of births especially of girls [31]. Our study along with the above two studies also highlights the challenges in evaluating such schemes. The challenges include lack of a control group, existing secular trend of development in the community with rapid changes in social structures and environment in last decade, absence of database that includes beneficiary status. Due to these, even if the analysis show a positive impact, it is very difficult to attribute changes to such schemes.

The strengths of this study include a comprehensive approach to the evaluation of girl child schemes through the use of multiple sources of information using a mixed methods approach and the use of longitudinal secondary data to measure its impact with both historic and current controls. The results from the three data sources as well as from different respondents reinforced each other. This triangulation adds confidence to our interpretation. The weaknesses include non availability of the data by beneficiary status and small number of interviews and the fact that the interviews were conducted by a medical doctor from a nearby hospital could have prejudiced the respondents during the indepth interviews. Also a more sophisticated statistical analysis adjusting for different confounders would have made the temporal trend assessment more robust. Our study area is different from rest of Haryana in terms of having a high coverage with antenatal care and vaccination and therefore, the improvement in overall coverage might have obliterated any sex differential. This indicates that strategies that improve the implementation of program to attain universal coverage may do better to address gender inequity than gender-specific programs. Overall, we feel that our study results are trustworthy and credible.

Overall, our study and other studies report a nil or modest degree of success of implementation of schemes which use CCTs to address larger social behaviors. One of the reasons for this could be that success of CCT programs require that these are set within a larger social protection system to ensure their financial sustainability; and to assure long-term institutional development. This is also reflected in a Government report on Gender and the Sex Ratio, which says “This situation poses a formidable challenge to public policy. It is not a phenomenon restricted to the very poor, which governments can attempt to solve through cash transfers or through the banning of medical diagnostic technologies alone. Clearly something is wrong, and successive governments have been unable to put their finger on the pulse of the problem. While it is difficult to tackle a problem that essentially stems from social and cultural attitudes as well as prejudices through State-led intervention *alone*, equally no nation can afford to *not* intervene when natural demography is tampered with in a manner that is unprecedented” [17].

Based on this study we make following recommendations:

1. Such schemes should not be seen as mere CCT schemes or subsidies but have to be a component of a multi-sectoral drive involving all stakeholders.

2. The schemes need to be revised if we want communities to change, to include universal eligibility, substantial increase in incentives and restructuring in such a way that it is useful for girl’s education and not only as a fund for marriage or dowry and finally to reduce the bureaucracy involved in its implementation.

## Competing interest

The authors declare that they have no competing interests.

## Authors’ contribution

KA conceived the study, analyzed the data, wrote the first draft of the paper. RA collected and analyzed the qualitative data. CSP, NN and PB provided critical inputs into the data analysis, interpretation and reviewed the manuscript. All authors read and approved the final manuscript.

## References

[B1] United Nations United Nations millennium declaration: resolution adopted by the General Assembly, 55th Session, 18 September 2000 2000 New York

[B2] United Nations The Millennium Development Goals Report 2011 2011 New York: United Nations

[B3] Zimmerman J Moury Y Savings-Linked Conditional Cash Transfers 2009 Washington DC: A New Policy Approach to Global Poverty Reduction. New America Foundation

[B4] Son HH Conditional Cash Transfer Programs: An Effective Tool for Poverty Alleviation? ERD policy brief no. 51 Asian Development Bank Manila 2008 July

[B5] Kornacki B “Conditional Cash Transfers: Progress Towards the Millennium Development Goals.” Sustainable Development Law & Policy, American University Washington College of Law Fall 2005 26

[B6] Rawlings LB Rubio GM Evaluating the Impact of Conditional Cash Transfer Programs The World Bank Research Observer, 20(1) 2005

[B7] Soares FV Silva E Conditional cash transfer programmes and gender vulnerabilities in Latin America Case studies from Brazil, Chile and Colombi Overseas Development Institute 2010 London October

[B8] Asadullah MN Chaudhury N Reverse gender gap in schooling in Bangladesh: insights from urban and rural households J Dev Stud 2009 45 8 1360 1380 10.1080/00220380902935824

[B9] Mayer P India’s falling sex ratios Popul Dev Rev 1999 25 2 323 343 10.1111/j.1728-4457.1999.00323.x

[B10] Murthi M Guio AC Dreze J Mortality, fertility and gender bias in India: a district-level analysis Popul Dev Rev 1995 21 4 745 782 10.2307/2137773

[B11] Census Commissioner of India. States at a glance 2012 New Delhi http://www.censusindia.gov.in/Census_Data_2001/States_at_glance/state_profile.aspx. Accessed on 13th May 2013

[B12] Krishnan A Ng N Byass P Pandav CS Kapoor SK Sex specific trends in under-five mortality in rural Ballabagrh Indian Pediatr 2013 [Epub ahead of print] 10.1007/s13312-014-0326-y 23999678

[B13] Krishnan A Dwivedi P Gupta V Byass P Pandav CS Ng N Socioeconomic development and girl child survival in rural North India: solution or problem? J Epidemiol Community Health 2013 67 5 419 426 10.1136/jech-2012-201846 23364028

[B14] Filmer D King EM Pritchett L Gender disparity in South Asia: comparisons between and within countries, Policy Research Working Paper 1867 1998 Washington, D.C: World Bank, Development Research Group

[B15] Sekher TV Special financial incentive schemes for the girl Child in India: a review of select schemes 2010 New Delhi: The Planning Commission, Government of India in collaboration with United Nations Population Fund

[B16] MODE Research Study on evaluation of Apni Beti Apna Dhan scheme in Haryana 2000 New Delhi: MODE Research Pvt Ltd July 2000.**nac**.nic.in/pdf/gsr_draft.pdf (Accessed on 11^th^ June 2013)

[B17] Naqvi F Shiva Kumar AK NAC Working Group on Gender & the Sex Ratio: Draft Recommendations 2012 Working Group of National Advisory Council on Gender and Declining Sex Ratio http://www.khubmarriage18.org/sites/default/files/129.pdf

[B18] Bardia A Paul E Kapoor SK Anand K Declining sex ratio: role of society technology and government regulation in Faridabad district, Haryana Natl Med J India 2004 17 4 207 211 15372767

[B19] Krishnan A Nongkynrih B Yadav K Singh S Gupta V Evaluation of computerized health management information system for primary health care in rural India BMC Health Serv Res 2010 10 310 10.1186/1472-6963-10-310 21078203 PMC2996385

[B20] Crabtree B Miller W Doing qualitative research 1999 Newbury Park, CA: Sage

[B21] Faherty VE Wordcraft: Applied Qualitative Data Analysis (QDA) Tools for Public and Voluntary Social Services 2010 London: Sage Publications

[B22] Nanda P Changing the value of girls in Haryana: Evaluation of the Apni Beti Apna Dhan CCT 2012 New Delhi: International Centre for Research on Women

[B23] Sharma R Goel R Gupta H Rajalakshmi – An Initiative for Improving the Status of Girl Child in Rajasthan J Fam Welf 2003 49 1 66 72

[B24] Srinivasan S Bedi AS Girl child protection scheme in Tamil Nadu: an appraisal Economic & Political Weekly 2009 48 10 12

[B25] Sekher T Ladlis and Lakshmis: financial incentive schemes for the girl child Economic & Political Weekly 2012 xlvii no 58 17 58 65

[B26] Sukhija P Delhi Laadli scheme: An appraisal. Working Paper No 239 2010 New Delhi: Centre for Civil Society

[B27] Nanda B The Ladli scheme in india: Leading to a Lehenga or a Law Degree? Department of Political Science. Miranda house Delhi university http://www.ipc-undp.org/pressroom/files/ipc126.pdf accessed on 13th May 2013

[B28] Paruthi R Sood AK A study on training needs assessment of panchayati raj members in the context of local decentralised development planning including health Health Popul Perspect Issues 1998 21 4 184 196

[B29] Sinha N Yoong J Long-Term Financial Incentives and Investment in Daughters Evidence from Conditional Cash Transfers in North India Policy Research Working Paper 4860 2009 The World Bank

[B30] Mazumdar CT Can Incentives Increase Preference for Daughters? Evidence from a Cash Transfer Scheme in Haryana, India The Georgetown public policy review 2012-13 18 1 64 80

[B31] Narasimhan RL Retherford RD Mishra V Arnold F Roy TK Comparison of Fertility Estimates from India’s Sample Registration System and National Family Health Survey. National Family Health Survey Subject Reports Number 4 • September 1997 Mumbai, India: International Institute for Population Sciences

